# Abdominal Tumors

**Published:** 1842-10

**Authors:** 


*Abdominal Tumors.*—It may seem strange that the diagnosis of abdominal tumors, which manifest themselves to the touch, and to the sight, should be so difficult and puzzling as it often is. I mentioned some reason for this before: the loose and shifting manner in which some of the viscera of the belly are packed and fastened; their liability to enlarge beyond their natural limits; their accidental dislocations under disease. It would be in vain to attempt even a sketch of the infinite variety of these deviations from the healthy state. Every case of abdominal tumor forms a separate object of study, and must be judged of by its proper circumstances. All that I can profess to do, is to offer you some rough hints on this interesting subject.

Some kinds of tumor result from morbid growths; such are all the varieties of cancer: some from the presence and multiplication of parasites; of which we have examples in collections of hydatids: some are produced by the distension of hollow organs; as where concretions, or fæcal matters, oras seg, lodge in the intestine: some consist in the mere enlargement of parts.

Let us enumerate the principal of these, that you may know what chiefly to expect.

1. There are, I say, tumors from lodgements in the *bowels;* and these are more hopeful than most kinds of abdominal tumors. Sometimes the stomach, or part of the intestinal canal, is distended in consequence of a mechanical impediment to the course of its contents: and this impediment may be invincible.

2. *Ovarian* tumors are very common. Of these I spoke at some length in a former lecture.

3. The *liver* is very liable to enlargement: either from simple congestion of blood; or from the interstitial deposit of adipous matter; or from the intrusion of malignant growths; or from colonies of hydatids.

4. So also the *spleen* swells, from fulness of blood. or from specific deposits in its substance.

5. The *kidneys* sometimes attain a vast size; being occupied by malignant disease, or swollen by pus that finds no vent.

6. Enlargements of the *mesenteric glands;* cancerous degeneration of the peritoneum, especially where it forms the *omentum;* tumors connected with the *uterus;* aneurisms of the *aorta;* constitute other species of abdominal swelling, which I simply mention without further comment.

Now our judgment of the character of a given tumor is naturally influenced by its *place*. In the right hypochondrium, we suspect the liver; in the left, the spleen; in the epigastric region, the stomach; in the hypogastric, the womb; in either flank, an ovary, or perhaps a kidney; in the track of the colon, we guess at fæcal collections.

But sometimes the situation of the tumor fits more than one, or than two, suppositions. Between the ribs and the ilium on the right side we may have an enlarged ovary, a tumid kidney, a distended cæcum. A prominence in the epigastrium may be due to cancer of the stomach, to an infarcted transverse colon, to a ventral aneurism. Above the pubes, the distended bladder, or the enlarged uterus, may equally project. The sigmoid flexure of the colon loaded with fæces, the left kidney exaggerated by disease, a bulky ovary, may either of them occupy the same sinistral space.

Moreover, the colon deviates strangely, and not seldom, from its natural course and position; and the magnified viscera may invade, by their displacement, or by their irregular expansion, the regions that are proper to other organs.

Our conjectures are assisted by the associated symptoms, and by observation of the regular performance, or of the disturbance, of particular functions. Yet here, also, we meet with continual sources of fallacy. Pressure from a tumor without may, as well as infarction within, impede the passage of alimentary matters through the bowels, of urine through the ureters; and cause, in the one case, flatulence and tormina—in the other, retention or suppression of urine. Growths foreign to the liver may, nevertheless, press upon its excretory ducts, and occasion jaundice. And so of other parts and func tions. I mean, that the functions prominently deranged are not always the functions of the part occupied by the tumor, but of organs which are secondarily and accidentally subjected to its disturbing influence. Your sagacity will be abundantly tried in balancing the evidence of different symptoms in these obscure, yet palpable, forms of disease: and after all, you will often doubt; and often, when you do not doubt, you will mistake.

Enlargement of the liver may usually be distinguished from other tumors of the right hypocondrium, by *percussion*. Try from the clavicle downwards. At first, you get a hollow sound. Then, a little below the nipple perhaps (for the spot varies much in different subjects), the sound begins to grow dull. If this dulness be traceable, without change or interruption, to the tumor, the inference is strong that the tumor is hepatic.

Any other tumor there situated leaves, most commonly, when the patient is recumbent, a palpable sulcus above it; or a space in which the sound, upon percussion, is different from that which is yielded by the liver.

Percussion helps us to discriminate an ovarian from a renal tumor. When the swelling is large, the intestines lie behind the one, in front of the other; and the sound is affected accordingly.

Tumors that are readily *moveable*, are generally intestinal, omental or ovarian.

A pulsating tumor is not necessarily an aneurism. The healthy artery will lift almost any sort of hard swelling that happens to lie directly over it.

The occurrence of hæmatemesis or melæna would corroborate your belief that a tumor in the right hypochondrium was hepatic—in the left, was splenic.

Even when you are satisfied as to the organ affected, there comes another question, scarcely, in some cases, less difficult than the first—What is the *nature* of the tumor?

Suppose, for the sake of illustration, that our inquiry relates to the liver. If the tumor be large, smooth, roundish, of slow growth, and the general health is not materially deranged, it is, most likely, an *hydatid* tumor. If along the edge and upon the surface of the augmented liver, you can feel large inequalities and projections, and if the complexion and general state of the patient are expressive of failing health, the enlargement is, in all probability, *cancerous:* and if there be other traces of carcinoma in the system, this conclusion becomes almost certain. Small, hard irregularities betoken the *hobnail* liver, which is sooner or later accompanied by ascites. When, without pain or jaundice, the liver of a phthisical patient transgresses its natural boundary, it is, in all probability, *fatty* liver.

By applying a similar method of investigation to other ventral enlargements, you may frequently hit the right scent, and trace the mischief to its true source. To treat the subject in detail would require a volume. I may refer you to a series of papers by Dr. Bright, in the Guy Hospital Reports; where you will find a host of examples, and much valuable information, concerning the most common and the most important kinds of "abdominal tumors and intumescence."—*Watson's Lectures.*

